# A systematic review of varicella seroprevalence in European countries before universal childhood immunization: deriving incidence from seroprevalence data

**DOI:** 10.1017/S0950268817001546

**Published:** 2017-08-22

**Authors:** K. BOLLAERTS, M. RIERA-MONTES, U. HEININGER, N. HENS, A. SOUVERAIN, T. VERSTRAETEN, S. HARTWIG

**Affiliations:** 1 P95 Pharmacovigilance and Epidemiology Services, Koning Leopold III Laan 1, Leuven 3001, Belgium; 2 Division of Paediatric Infectious Diseases and Vaccinology, University of Basel Children's Hospital, Basel CH-4056, Switzerland; 3 Interuniversity Institute for Biostatistics and statistical Bioinformatics, Hasselt University, Diepenbeek, Belgium; 4 Centre for Health Economics Research and Modelling Infectious Diseases and Centre for the Evaluation of Vaccination, Vaccine & Infectious Disease Institute, University of Antwerp, Antwerp, Belgium; 5 Aixial, 4 rue Danjou, Boulogne-Billancourt 92513, France; 6 Sanofi Pasteur MSD, 162 avenue Jean Jaurès, Lyon 69007, France

**Keywords:** Chickenpox, Europe, immunization, incidence, seroepidemiology, varicella zoster virus

## Abstract

Surveillance systems for varicella in Europe are highly heterogeneous or completely absent. We estimated the varicella incidence based on seroprevalence data, as these data are largely available and not biased by under-reporting or underascertainment. We conducted a systematic literature search for varicella serological data in Europe prior to introduction of universal varicella immunization. Age-specific serological data were pooled by country and serological profiles estimated using the catalytic model with piecewise constant force of infection. From the estimated profiles, we derived the annual incidence of varicella infection (/100·000) for six age groups (<5, 5–9, 10–14, 15–19, 20–39 and 40–65 years). In total, 43 studies from 16 countries were identified. By the age of 15 years, over 90% of the population has been infected by varicella in all countries except for Greece (86·6%) and Italy (85·3%). Substantial variability across countries exists in the age-specific annual incidence of varicella primary infection among the <5 years old (from 7052 to 16 122 per 100 000) and 5–9 years old (from 3292 to 11 798 per 100 000). The apparent validity and robustness of our estimates highlight the importance of serological data for the characterization of varicella epidemiology, even in the absence of sampling or assay standardization.

## INTRODUCTION

Varicella (chickenpox) is a common, vaccine-preventable disease, caused by a double-stranded DNA virus of the herpesvirus family, varicella zoster virus (VZV) [1]. Varicella is highly contagious and infection in the pre-vaccine era is almost universal [2]. Varicella is usually a mild disease occurring in early childhood, but complications can occur. The risk of complications increases with age [2]. After primary infection, varicella becomes latent in dorsal root and cranial nerve ganglia. Reactivation of the latent virus due to waning immunity leads to herpes zoster (shingles), a disease which affects dermatomes corresponding to the site of viral reactivation [3].

Epidemiological data are essential for priority setting and planning of immunization strategies. However, varicella surveillance is rarely carried out systematically in Europe, with most European countries using data from either mandatory reporting or sentinel surveillance sites [4]. Varicella is not included in the European Union (EU) list of mandatory reportable diseases, and consequently there is no standardized case definition for reporting [5]. As European surveillance systems vary in terms of cases captured (all cases *vs*. cases with complications), case validation methods (e.g. clinical, laboratory, or epidemiologically-linked) and data granularity (i.e. case-based or aggregated data), obtaining consistent data of the burden of disease of varicella in European countries is challenging [5]. In addition, these surveillance systems are passive and subject to under-reporting [6, 7]. The degree of underascertainment can also be considerable since clinical surveillance only captures medically attended disease, while varicella patients frequently do not seek medical care [8]. Indeed, varicella disease is often mild (especially in young children) and, depending on the country-specific health care-seeking practices, these patients often do not seek health care.

In contrast, varicella seroprevalence studies [9] in the absence of immunization measure all previous infections (asymptomatic, medically attended disease, or not medically attended disease) as varicella antibodies are lifelong. Seroprevalence studies are therefore not prone to underascertainment and under-reporting biases. Such studies are frequently conducted for various childhood vaccine preventable diseases [10–12], including varicella [9], and are used to estimate infectious disease transmission parameters, such as the basic reproduction number R_0_ and the force of infection [9, 13]. Only few examples of estimating varicella incidence based on serological data in Europe have been published (Luxembourg [14], Spain [15] and Italy [16]).

We carried out a systematic literature review of varicella studies before the introduction of universal varicella immunization in Europe with the objective of estimating the burden of varicella infections [17]. Here we report the results on the seroprevalence data. Specifically, to deal with the heterogeneity in age groups of the reported seroprevalence data, we first estimate country-specific seroprevalence profiles in function of age, based on which we derive the annual age-specific incidence of primary VZV infection (per 100 000). This is, to our knowledge, the first systematic literature review of European studies on VZV-IgG antibody seroprevalence and the first study to report age-specific varicella incidence rates derived from seroprevalence data across Europe.

## METHODS

### Systematic literature review

#### Data sources

A PubMed search was conducted for peer-reviewed publications in any language using the search string ‘Varicella AND (mortality OR complications OR epidemiology OR seroprevalence OR prevalence OR incidence)’ with the search limited to member states of the EU (Austria, Belgium, Bulgaria, Croatia, Republic of Cyprus, Czech Republic, Denmark, Estonia, Finland, France, Germany, Greece, Hungary, Ireland, Italy, Latvia, Liechtenstein, Lithuania, Luxembourg, Malta, the Netherlands, Poland, Portugal, Romania, Slovakia, Slovenia, Spain, Sweden and the UK), Norway, Iceland and Switzerland. Additional information was obtained from the European Center for Disease Prevention and Control (ECDC) website, from national health institutes websites and through personal communication with country-level varicella surveillance ECDC focal points. Hand searching of the reference lists of papers selected for inclusion was conducted to identify additional publications.

#### Study selection

Serological studies were eligible for inclusion if the data were collected before the introduction of universal varicella immunization in the country's immunization program (year of introduction as documented in reference [18]), were published on or after 1 January 1995 and provided sufficient information to be included in the analyses (sample size or 95% confidence interval). Studies on specific subgroups (e.g. day-care workers, prisoners, individuals with a negative varicella history) were excluded. Studies that did not provide data for at least one pediatric age group <9 years were included only if there were additional data sources from the same country that included pediatric estimates. Two reviewers (MR-M and MB) screened titles and abstracts. Discrepancies were extensively discussed and no third reviewer was necessary to resolve disagreements. Full-text eligibility was evaluated by a single reviewer (MR-M).

#### Data collection process

Data extraction from selected studies was performed by a single reviewer (MR-M) into an Excel output file. The following data were extracted: author, journal, year of publication, country, population, age range and by age group; sample size, number of subjects that tested positive, varicella seroprevalence and 95% confidence interval. Additional information was obtained on laboratory methods, commercial tests and handling of indeterminate test results. As quality control, a sample of 10% of the papers was re-extracted by a second reviewer (TV).

### Statistical analyses

Varicella seroprevalence data were pooled within each country after visually checking for outlying age profiles. Data on age groups including children below 6 months of age were discarded as seropositivity under that age is confounded with persisting maternal antibodies [19]. The pooled data were then used to model the country- and age-specific seroprevalence *π*(*a*) as a function of midage. To estimate *π*(*a*), we used the catalytic model with a piecewise constant force of infection *λ*(*a*) (or the rate at which susceptible individuals acquire the infection per unit time) as used in Nardone *et al.* [9]. As nearly all children are seropositive by late childhood, this model estimates the force of infection for three age groups: <5, 5–9 and ⩾10 years of age, assuming lifelong immunity, time homogeneity and non-differential mortality [9]. We used maximum likelihood ensuring monotonicity through restricting the parameter space.

We additionally modeled the age-specific seroprevalence using isotonic splines regression [20, 21] to verify whether the catalytic model with piecewise constant force of infection is sufficiently flexible. If this was the case, we opted to derive the incidence from the catalytic model. We deemed the latter model preferable to the isotonic splines model because it provides an easier extrapolation to age ranges for which there are no observations as well as direct estimates of the forces of infection.

Finally, starting from the estimated seroprevalence, we derived the annual age-specific incidence of primary VZV infection (or the number of new infections within the total population) from differences in seroprevalences (see Section ‘Deriving incidence from seroprevalence’ for the rationale of this approach). In particular, we derived the annual average incidence (per 100 000) for the age groups: <5, 5–9, 10–14, 15–19, 20–39 and 40–65 years of age. Bootstrapping was used to obtain the 95% percentile confidence intervals of the age-specific incidences and forces of infection. All analyses were carried out in R 3·01 [22].

### Deriving incidence from seroprevalence

Typically, the incidence (measure of infection in the total population) is derived by multiplying an estimate of the force of infection (measure of infection in the susceptible population) with an estimate of the proportion of susceptibles within the population [23]. Indeed, given the age-dependent force of infection *λ*(*a*) = *x*(*a*)/*s*(*a*) – with *x*(*a*) being the number of new cases of age *a* per unit time and *s*(*a*) being the number of susceptibles at age *a* – and given the age-specific incidence *i*(*a*) = *x*(*a*)/*n*(*a*) – with *n*(*a*) being the population size at age *a* – it follows that the incidence per unit time at age *a* equals:
1
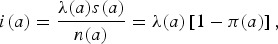

with [1 − *π*(*a*)] = *s*(*a*)/*n*(*a*) being the proportion of susceptibles within the population at age *a*.

The force of infection *λ* can be derived from the age-specific prevalence *π*(*a*) under the assumptions of lifelong immunity, time homogeneity and non-differential mortality [24]. As early as in the 1930s, Muench [25] introduced the catalytic model, assuming an exponential depletion of susceptibles at a constant rate *λ* or
2
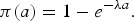


The assumption of an age-independent force of infection is not always appropriate and has led to several model extensions allowing the force of infection to vary with age [20, 26–32]. The only requirement of a consistent model is that the force of infection is non-negative, or that *π*(*a*) is a non-decreasing function of age. This is not an intrinsic property of the models above, but can be guaranteed by restricting the parameter space [27, 32] or by imposing monotonicity constraints [20, 31].

When the force of infection depends on age, expression (2) becomes
3
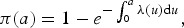

with *λ*(*a*) being the age-specific force of infection. Given the initial condition that *π*(0) = 0 (meaning that everyone is susceptible at birth), expression (3) is the solution to the differential equation
4
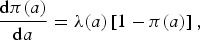

describing the change in the non-susceptible fraction *π*(*a*) = 1 − *s*(*a*)/*n*(*a*) over age [26]. As such, the force of infection equals
5
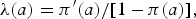

with *π*′(*a*) being the first derivative of *π*(*a*) with respect to age *a*. From substituting (5) in (1), it follows that the incidence rate at age *a* is
6
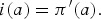


Then, the cumulative incidence from age *a* to *a* + *k* equals
7
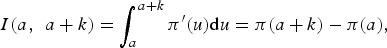

which is simply a difference in seroprevalences. Finally, assuming a constant incidence within the age interval [*a*,   *a* + *k*], we obtain the average annual incidence rate (/100·000) through dividing the cumulative incidence for the age interval by the width of the interval:
8
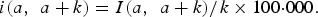


## RESULTS

### Selected studies

**Fig. 1.**
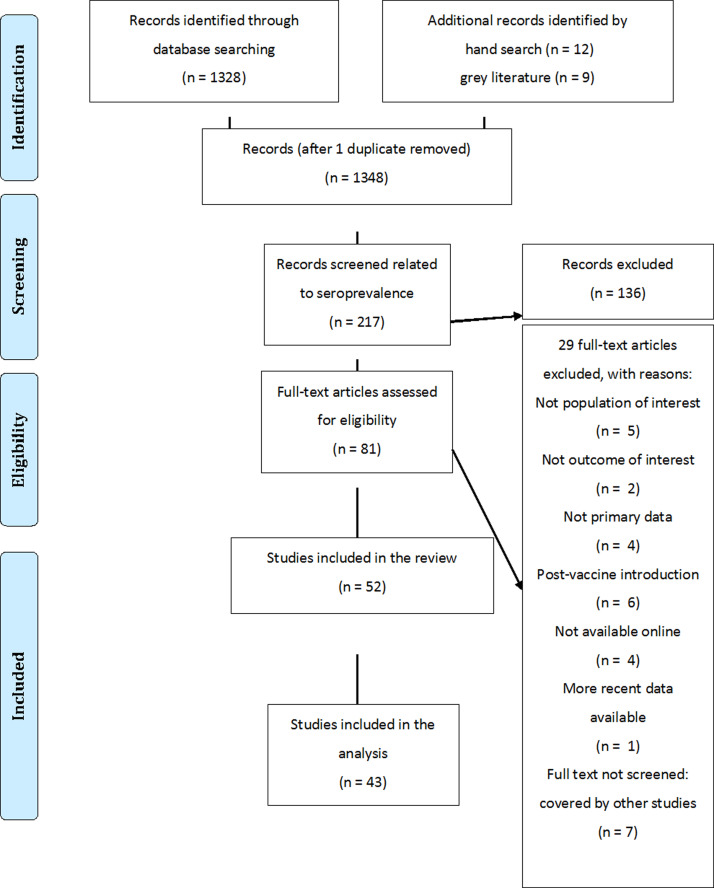
Study selection (PRISMA flow diagram).

The PubMed search was conducted on 2 October 2015. The results of the literature search are shown as a PRISMA diagram in Figure 1. We identified 52 seroprevalence studies from 20 countries. Of these, two were excluded from further analyses because of data duplication with another source (UK and the Netherlands), two because they did not provide age range or mean age (Ireland and Spain) and five because no data for children <9 years of age were available at country level (Austria, Portugal (2), Sweden and Croatia). The characteristics of the remaining 43 studies coming from 16 European countries are summarized in online Supplementary Table S1.

### Seroprevalence

**Fig. 2.**
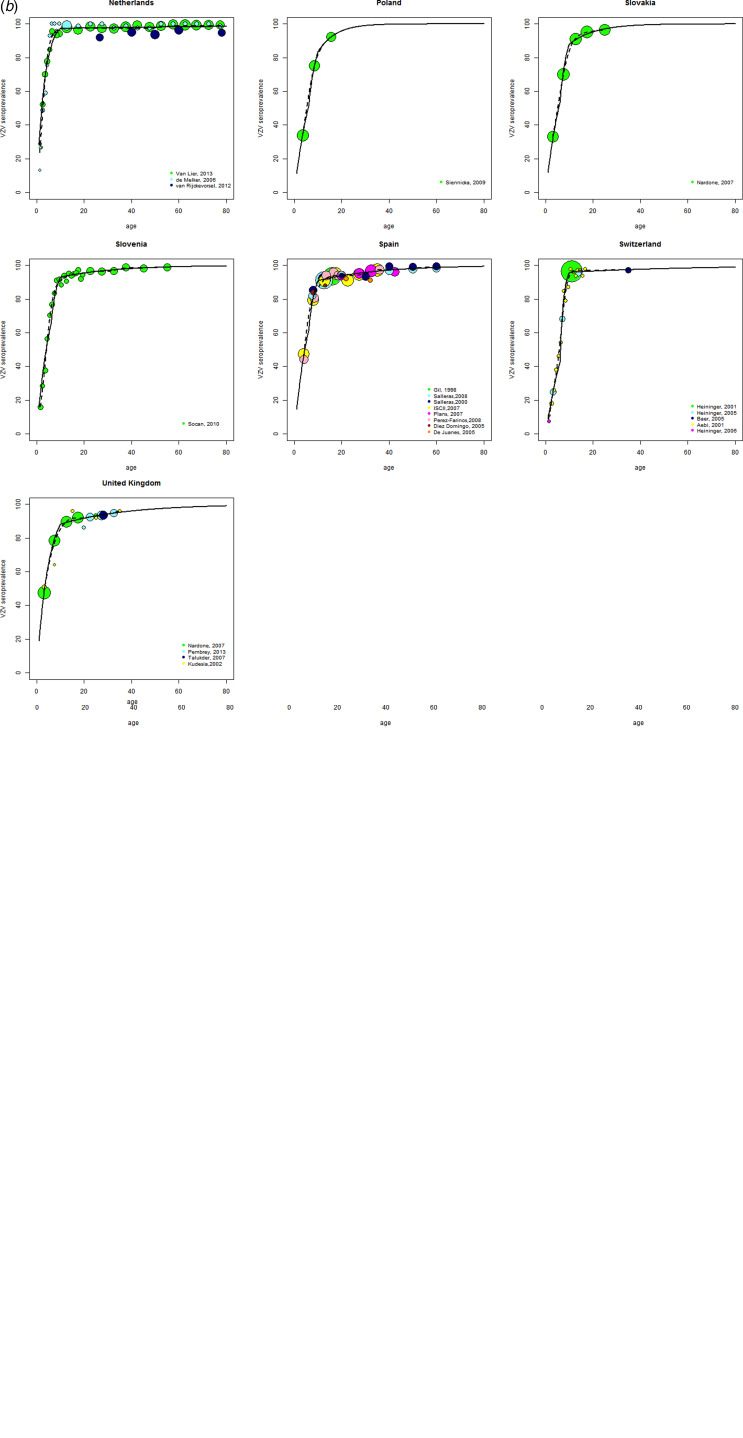
Observed age-specific VZV seroprevalence (circles with the area reflecting the sample size) and age-specific seroprevalence profiles as estimated by the catalytic model with piecewise constant force of infection (solid line) and the isotonic splines model (dashed line) before universal childhood immunization, by country.

The results were consistent within the different countries for which more than one study was available, with the exception of Italy. For Italy, one study was excluded because it showed an outlying age profile compared with the other Italian studies, an observation also made by the authors of the study [33]. For all countries, the catalytic model and the isotonic splines model provided very similar and good fits to the data with *R*^2^ values >0·85 for all countries (Fig. 2). The catalytic model was therefore preferred. The age-specific force of infection estimates are provided in online Supplementary Table S2. The serological profiles showed a VZV-IgG seroprevalence of over 80% by the age of 10 years for all countries except for Greece and 90% by the age of 15 years for all countries except for Greece (86·6%) and Italy (85·3%) (Table 1). VZV-IgG seroprevalence data suggest that the great majority of children and adolescents seroconvert before adulthood. The age at which this happens varies, with substantial between-country differences in the seroprevalences in the <5 and 5–9 years old.

**Table 1.** Age-specific seroprevalence (%) of varicella in 16 European countries before the introduction of universal childhood immunization programs

	Seroprevalence (%) (95% CI) per age group (years)					
	<5	<10	<15	<20	<40	<65
Belgium	73·1 (69·2–77·0)	93·8 (91·2–95·8)	95·2 (93·6–96·5)	96·3 (95·3–97·1)	98·7 (98·4–99·0)	99·6 (99·4–99·8)
Finland	50·7 (46·7–54·4)	94·1 (92·8–95·0)	94·2 (93·3–95·1)	94·4 (93·7–95·2)	95·1 (94·5–96·0)	95·9 (94·7–97·2)
France	67·4 (63·4–71·4)	90·8 (87·2–93·9)	93·6 (91·7–95·4)	95·5 (94·2–96·8)	98·9 (97·9–99·5)	99·8 (99·3–100)
Germany	59·4 (55·3–63·2)	94·7 (93·3–95·8)	95·9 (95·0–96·7)	96·8 (96·2–97·5)	98·9 (98·5–99·3)	99·7 (99·4–99·9)
Greece	35·3 (29·9–40·7)	72·6 (61·3–80·6)	86·6 (72·1–97·6)	93·4 (72·7–99·8)	99·6 (72·9–100)	100 (73·0–100)
Iceland	57·3 (42·0–62·6)	96·9 (76·2–100)	100 (100–100)	100 (100–100)	100 (100–100)	100 (100–100)
Ireland	59·8 (53·7–66·3)	91·9 (88·8–93·5)	92·3 (90·6–93·6)	92·7 (91·6–93·7)	94·0 (91·9–96·9)	95·3 (91·9–98·9)
Italy	40·1 (36·5–43·5)	80·7 (78·0–83·2)	85·3 (83·9–86·6)	88·8 (88·0–89·6)	96·2 (95·1–97·1)	99·0 (98·3–99·4)
Luxembourg	78·6 (72·6–84·3)	95·1 (93·5–96·4)	95·8 (94·8–96·8)	96·5 (95·7–97·3)	98·2 (97·3–99·0)	99·2 (98·3–99·7)
The Netherlands	80·6 (78·2–83·0)	97·4 (96·7–98·0)	97·5 (96·9–98·0)	97·6 (97·1–98·0)	97·9 (97·6–98·2)	98·3 (97·9–98·7)
Poland	44·9 (40·2–49·5)	83·5 (78·59–87·6)	91·8 (89·0–94·3)	95·9 (92·3–98·0)	99·7 (97·7–100)	100 (99·5–100)
Slovakia	46·8 (42·0–51·9)	88·1 (84·9–90·9)	92·5 (91·3–93·8)	95·3 (94·0–96·5)	99·3 (98·1–99·8)	99·9 (99·5–100)
Slovenia	58·2 (53·7–62·2)	93·0 (91·1–94·5)	94·3 (93·2–95·4)	95·4 (94·6–96·3)	98·1 (97·1–98·9)	99·3 (98·5–99·8)
Spain	54·4 (50·8–58·1)	90·9 (90·0–91·9)	92·5 (91·9–93·1)	93·8 (93·3–94·3)	97·1 (96·6–97·6)	98·9 (98·4–99·3)
Switzerland	36·8 (32·0–41·3)	95·8 (94·9–96·5)	96·2 (95·5–97·1)	96·5 (95·6–98·1)	97·6 (95·7–99·6)	98·5 (95·8–99·9)
UK	64·9 (60·8–68·6)	88·2 (85·5–90·7)	90·1 (88·6–91·7)	91·8 (90·6–92·9)	96·0 (94·0–97·5)	98·4 (96·1–99·4)

95% CI, 95% confidence interval.

### Incidence

**Fig. 3.**
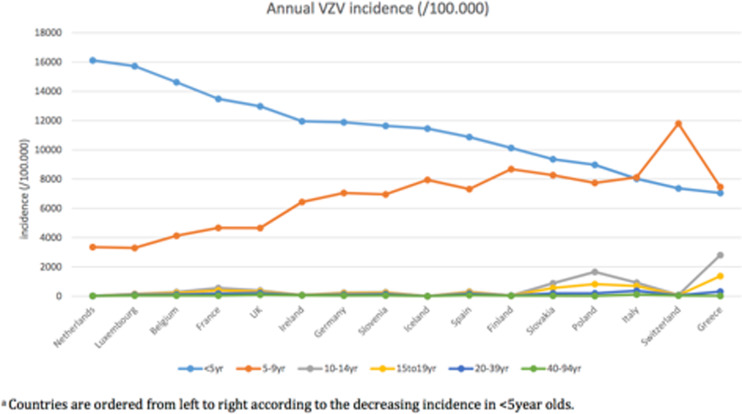
Age-specific annual incidence (/100·000) of VZV in sixteen European countries* before the introduction of universal childhood immunization programs by age group.

The highest incidence rates were observed among the <5 years age group in all countries except for Italy, Switzerland and Greece (Fig. 3 and Table 2). Age-specific annual incidence rates of primary varicella infection varied considerably across the countries from 7052/100 000 (Greece) to 16 122 (the Netherlands) for the <5 years old and from 3292/100 000 (Luxembourg) to 11 798/100 000 (Switzerland) for the 5–9 years old. Generally, the incidence in the 5–9 years old increases with decreasing incidence in the <5 years old (Fig. 3). Primary varicella infection incidence rates drop drastically from 10 years of age onwards with the highest mean estimates in the 10–14 years old in Poland (1652/100 000), in the 15–19 years old in Greece (1370/100 000), and in the 20–39 and 40–64 years old in Italy (371·5 and 112·8/100 000, respectively). Generally, for countries with a high incidence in the <5 years old (i.e. incidence of 10 000/100 000 or more), the incidence in the age groups 10 years and above is very low (Fig. 3).

**Table 2.** Age-specific annual incidence rate (/100·000) of VZV primary infection in 16 European countries before the introduction of universal childhood immunization programs

	Annual incidence (/100·000) (95% CI) per age group (years)					
	<5	5–9	10–14	15–19	20–39	40–64
Belgium	14 628 (13 872–15 340)	4126 (3206–4982)	284 (130–484)	220 (110–346)	119 (71–165)	38 (31–45)
Finland	10 130 (9354–10 872)	8680 (7882–9516)	40 (0–106)	38 (0–98)	35 (0–80)	30 (0–56)
France	13 488 (12 658–14 252)	4668 (3506–5812)	554 (250–980)	388 (202–594)	171·5 (117–221)	36 (16–56)
Germany	11 884 (11 070–12 702)	7048 (6104–7952)	246 (152–384)	190 (126–270)	103 (77–129)	32 (22–40)
Greece	7052 (5958–8142)	7462 (4814–9580)	2804 (0–6424)	1370 (0–1694)	309 (0–555)	14 (0–218)
Iceland	11 460 (8400–12 520)	7940 (6840–11 260)	0 (0–0)	0 (0–0)	0 (0–0)	0 (0–0)
Ireland	11 954 (10 730–13 152)	6434 (4946–7768)	76 (0–426)	72 (0–338)	64 (0–208)	52 (0–90)
Italy	8020 (7290–8716)	8118 (7002–9168)	916 (644–1292)	698 (524–918)	371 (313–427)	112 (94–127)
Luxembourg	15 720 (14 456–16 838)	3292 (2080–4–662)	152 (52–300)	128 (48–230)	86 (40–123)	41 (28–49)
The Netherlands	16 122 (15 662–16 580)	3350 (2872–3830)	20 (2–46)	20 (2–42)	18 (2–36)	15 (2–26)
Poland	8974 (7980–9990)	7734 (6134–9148)	1652 (780–2670)	822 (534–1022)	191 (94–285)	9 (0·8–63)
Slovakia	9362 (8392–10 354)	8264 (6880–9520)	882 (464–1482)	554 (346–756)	198 (156–230)	26 (9–57)
Slovenia	11 640 (10 756–12 466)	6954 (5952–7944)	274 (142–452)	220 (122–334)	132 (88–168)	51 (35–60)
Spain	10 874 (10 158–11 570)	7312 (6532–8078)	314 (238–398)	260 (202–318)	165 (139–188)	71 (64–77)
Switzerland	7368 (6488–8324)	11 798 (10 816–12 688)	74 (0–318)	68 (0–210)	54 (0–93)	36 (0–40)
UK	12 982 (12 226–13 780)	4656 (3652–5632)	388 (160–662)	324 (146–506)	211 (117–275)	95 (73–108)

95% CI, 95% confidence interval.

## DISCUSSION

This systematic review with re-analyses of the serological data provides a comprehensive overview of the seroepidemiology of varicella in Europe prior to the introduction of universal varicella immunization. In total, 43 studies from 16 European countries were used for the analyses. Despite the differences in study characteristics (e.g. study populations, study periods, laboratory tests, handling of equivocal test results), VZV-IgG seroprevalence results were found to be consistent within countries, confirming the robustness of the serological data. In all countries, the vast majority of VZV-IgG antibody acquisition occurred in children under 10 years old, but inter-country variability in the speed of acquisition was observed (Table 1).

VZV-IgG seroprevalence data suggest that nearly all people seroconvert before adulthood. The age at which this happens varies, resulting in three clusters of European countries. The first group represents those with earliest exposure where seroprevalence values already reach 70% or more by the age of 5 years (Belgium, Luxemburg and the Netherlands). The second group reaches <70% seroprevalence by the age of 5 years but over 90% by the age of 10 years (Finland, France, Germany, Iceland, Ireland, Slovenia, Spain and Switzerland). Finally, the group of the latest exposure does not reach 90% seroprevalence by the age of 10 years (Greece, Italy, Poland, Slovakia and the UK). With the exception of the first cluster, these clusters are not geographically co-located, suggesting that other factors than geography play a role in these differences. Differences in social mixing due to micro (i.e. the household-level) and macro (i.e. the community) population structures may influence the age of VZV infection [34–36]. At the community level, differences in early child care [36–38], population density, inequality in wealth and infant vaccination coverage [36] have all been observed to be directly or indirectly related to varicella transmission and might explain the observed inter-country variation.

Derived varicella incidences were highest among children <5 years of age in all countries except for Italy, Switzerland and Greece, where the highest mean varicella incidences were observed in 5–9 years old. The annual varicella incidence ranged from 7052/100 000 (Greece) to 16 122/100 000 (the Netherlands) among <5 years old, and from 3292/100 000 (Luxembourg) to 11 798/100 000 (Switzerland) in 5–9 years old.

The approach of deriving varicella incidence from age-specific cross-sectional VZV-IgG seroprevalence data has limitations. It requires the assumption of lifelong immunity, time homogeneity and non-differential mortality. We deemed time homogeneity to be the strongest assumption. Varicella does not have a pronounced epidemic cycle but its incidence tends to fluctuate in 2–5 years cycles [39]. Although we did not account for cyclical patterns in childhood diseases, this has previously been shown to have little effect when estimating common infectious disease parameters from seroprevalence data [40]. In addition, by comparing and pooling data across different studies, we assumed representative study populations and equivalent serum testing methodology. However, some studies used residual serum samples, while others used population-based random sampling. In addition, equivocal results were not dealt with uniformly across studies: some studies exclude them, others re-test and others classify them as positive or negative (online Supplementary Table S1). However, we observed a strong intra-country consistency of the different studies with only one study being excluded because of an outlying age profile. This suggests that the impact of differences in study populations and serum testing methodology is small.

Our results are consistent with a previous VZV infection seroepidemiology study that was conducted in 11 European countries by Nardone *et al.* [9], which was also included in our analyses. As with our study, Nardone *et al.* found that the vast majority of virus transmission occurred in children <10 years of age while substantial differences in the speed of VZV-IgG acquisition were observed between countries (seroprevalence in the <5 years old ranged from 38% in Italy to 97% in the Netherlands).

Our estimated varicella incidence rates were generally higher than the estimates reported from sentinel surveillance networks and mandatory notification [41–50]. The biggest differences were seen in Belgium, the Netherlands and Italy. Based on the serological data, we estimated an annual incidence in children <5 years of age of 14 600 (Belgium), 16 100 (the Netherlands) and 8000 (Italy) per 100 000. Corresponding estimates based on sentinel surveillance were between 4400 and 5500 annual cases per 100 000 for Belgium [41], between 3000 and 6100 for the Netherlands [49, 50] and between 1350 and 3900 for Italy. These differences are probably explained by under-reporting [16] and by different patterns of health care utilization in different countries [49].

This is, to our knowledge, the first study to report age-specific varicella incidence rates derived from seroprevalence data across Europe. We showed that the age-specific incidence rates can be easily derived from seroprevalence data as the difference between age-specific seroprevalences. We found the highest incidence rates among children <5 years of age in all countries except for Italy, Switzerland and Greece. Our estimates appear to be valid and robust even in the absence of sampling or assay standardization. These findings highlight the importance of serological data from the pre-vaccination period for obtaining a comparable characterization of VZV epidemiology across European countries, since they are, unlike incidence estimates directly obtained from surveillance, not affected by underascertainment and under-reporting.

In subsequent work [18], the varicella incidence was used as one of the components to estimate the total burden of varicella by different levels of severity (cases in the community, health care seekers in primary care and hospitals, and deaths). Such detailed burden of disease estimates is essential for the planning and evaluation of varicella control strategies.

## Supplementary material

For supplementary material accompanying this paper visit http://dx.doi.org/10.1017/S0950268817001546. click here to view supplementary material

## References

[1] Arvin AM. Varicella-zoster virus. Clinical Microbiology Reviews 1996; 9(3): 361–381. 8809466 10.1128/cmr.9.3.361 PMC172899

[2] Helmuth IG, Varicella in Europe-a review of the epidemiology and experience with vaccination. Vaccine 2015; 33(21): 2406–2413. 25839105 10.1016/j.vaccine.2015.03.055

[3] Sauerbrei A. Diagnosis, antiviral therapy, and prophylaxis of varicella-zoster virus infections. European Journal of Clinical Microbiology & Infectious Diseases 2016; 35(5): 723–734. 26873382 10.1007/s10096-016-2605-0

[4] Anon. EUVAC.NET. Surveillance of Varicella and Herpes Zoster in Europe, November 2010. (http://www.euvac.net/graphics/euvac/pdf/varicella_zoster_surveillance.pdf).

[5] Gauthier A, Epidemiology and cost of herpes zoster and post-herpetic neuralgia in the United Kingdom. Epidemiology & Infection 2009; 137(1): 38–47. 18466661 10.1017/S0950268808000678

[6] Bonhoeffer J, Prospective surveillance of hospitalisations associated with varicella-zoster virus infections in children and adolescents. European Journal of Pediatrics 2005; 164(6): 366–370. 15747132 10.1007/s00431-005-1637-8

[7] Socan M. Evaluation of mandatory case-based reporting system for varicella in the prevaccine era. Central European Journal of Public Health 2010; 18(2): 99–103. 20939260 10.21101/cejph.a3572

[8] Fleming DM, The incidence of chickenpox in the community. Lessons for disease surveillance in sentinel practice networks. European Journal of Epidemiology 2001; 17(11): 1023–1027. 12380716 10.1023/a:1020066806544

[9] Nardone A, The comparative sero-epidemiology of varicella zoster virus in 11 countries in the European region. Vaccine 2007; 25(45): 7866–7872. 17919788 10.1016/j.vaccine.2007.07.036

[10] de Melker H, The seroepidemiology of measles in Western Europe. Epidemiology & Infection 2001; 126(2): 249–259. 11349976 10.1017/s0950268801005234 PMC2869690

[11] Edmunds WJ, The sero-epidemiology of diphtheria in Western Europe. ESEN project. European Sero-Epidemiology Network. Epidemiology & Infection 2000; 125(1): 113–125. 11057967 10.1017/s0950268899004161 PMC2869577

[12] Pebody RG, The seroepidemiology of rubella in Western Europe. Epidemiology & Infection 2000; 125(2): 347–357. 11117958 10.1017/s0950268899004574 PMC2869607

[13] Edmunds WJ, The pre-vaccination epidemiology of measles, mumps and rubella in Europe: implications for modelling studies. Epidemiology & Infection 2000; 125(3): 635–650. 11218214 10.1017/s0950268800004672 PMC2869647

[14] Mossong J, Putz L, Schneider F. Seroprevalence and force of infection of varicella-zoster virus in Luxembourg. Epidemiology & Infection 2004; 132(6): 1121–1127. 15635970 10.1017/s0950268804002754 PMC2870204

[15] Munoz MP, Dominguez A, Salleras L. Estimated varicella incidence on the basis of a seroprevalence survey. Epidemiology & Infection 2001; 127(3): 501–507. 11811884 10.1017/s0950268801006264 PMC2869776

[16] Ciofi degli Atti ML, Assessment of varicella underreporting in Italy. Epidemiology & Infection 2002; 128(3): 479–484. 12113493 10.1017/s0950268802006878 PMC2869845

[17] Riera-Montes M, Estimation of the burden of varicella in Europe before the introduction of universal childhood immunization. BMC Infectious Diseases 2017; 17(1): 353. 28521810 10.1186/s12879-017-2445-2 PMC5437534

[18] Anon. European Centre for Disease Prevention and Control (ECDC). ECDC guidance: Varicella vaccination in the European Union, 2015 (http://ecdc.europa.eu/en/publications/Publications/Varicella-Guidance-2015.pdf).

[19] Heininger U, Desgrandchamps D, Schaad UB. Seroprevalence of Varicella-Zoster virus IgG antibodies in Swiss children during the first 16 months of age. Vaccine 2006; 24(16): 3258–3260. 16459000 10.1016/j.vaccine.2006.01.026

[20] Hens N, Modeling Infectious Disease Parameters Based on Serological Data and Social Contact Data. London: Springer, 2012.

[21] Bollaerts K, Eilers PH, van Mechelen I. Simple and multiple P-splines regression with shape constraints. British Journal of Mathematical and Statistical Psychology 2006; 59(Pt 2): 451–469. 17067421 10.1348/000711005X84293

[22] R Core Team. R: A language and environment for statistical computing. R Foundation for Statistical Computing, Vienna, Austria, 2013 (http://www.R-project.org/).

[23] Vynnycky E, White RG. An Introduction to Infectious Disease Modelling. Oxford: Oxford University Press, 2010.

[24] Anderson RM, May RM. Directly transmitted infections diseases: control by vaccination. Science 1982; 215(4536): 1053–1060. 7063839 10.1126/science.7063839

[25] Muench H. Derivation of rates from summation data by the catalytic curve. Journal of the American Statistical Association 1959; 29(185): 14.

[26] Hens N, Seventy-five years of estimating the force of infection from current status data. Epidemiology & Infection 2010; 138(6): 802–812. 19765352 10.1017/S0950268809990781

[27] Farrington CP. Modelling forces of infection for measles, mumps and rubella. Statistics in Medicine 1990; 9(8): 953–967. 2218197 10.1002/sim.4780090811

[28] Gabutti G, The epidemiology of Varicella Zoster Virus infection in Italy. BMC Public Health 2008; 8: 372. 18954432 10.1186/1471-2458-8-372 PMC2601043

[29] Grenfell BT, Anderson RM. The estimation of age-related rates of infection from case notifications and serological data. Journal of Hygiene *(*London*)* 1985; 95(2): 419–436. 10.1017/s0022172400062859 PMC2129533 4067297

[30] Griffiths D. A catalytic model of infection for measles. Applied Statistics 1974; 23: 10.

[31] Keiding N, Estimation from current-status data in continuous time. Lifetime Data Analysis 1996; 2(2): 119–129. 9384639 10.1007/BF00128570

[32] Shkedy Z, Modelling age-dependent force of infection from prevalence data using fractional polynomials. Statistics in Medicine 2006; 25(9): 1577–1591. 16252265 10.1002/sim.2291

[33] Alfonsi V, Susceptibility to varicella in childbearing age women, Central Italy: is there a need for vaccinating this population group? Vaccine 2007; 25(32): 6086–6088. 17629374 10.1016/j.vaccine.2007.05.019

[34] Yu AL, Three year seroepidemiological study of varicella-zoster virus in Sao Paulo, Brazil. Revista do Instituto de Medicina Tropical de Sao Paulo 2000; 42(3): 125–128. 10887369 10.1590/s0036-46652000000300002

[35] Silhol R, Micro and macro population effects in disease transmission: the case of varicella. Epidemiology & Infection 2010; 138(4): 482–490. 19796448 10.1017/S0950268809990896

[36] Santermans E, The social contact hypothesis under the assumption of endemic equilibrium: elucidating the transmission potential of VZV in Europe. Epidemics 2015; 11: 14–23. 25979278 10.1016/j.epidem.2014.12.005

[37] Kudesia G, Changes in age related seroprevalence of antibody to varicella zoster virus: impact on vaccine strategy. Journal of Clinical Pathology 2002; 55(2): 154–155. 11865016 10.1136/jcp.55.2.154 PMC1769589

[38] Brisson M, Epidemiology of varicella zoster virus infection in Canada and the United Kingdom. Epidemiology & Infection 2001; 127(2): 305–314. 11693508 10.1017/s0950268801005921 PMC2869750

[39] Seward J, Jumaan A. VSV: persistence in the population. In: Arvin A, , eds. Human Herpesviruses: Biology, Therapy, and Immunoprophylaxis (pp. 713–734). Cambridge: Cambridge: Cambridge University Press, 2007. 10.1017/CBO9780511545313.041. 21348071

[40] Whitaker HJ, Farrington CP. Estimation of infectious disease parameters from serological survey data: the impact of regular epidemics. Statistics in Medicine 2004; 23(15): 2429–2443. 15273957 10.1002/sim.1819

[41] Sabbe M, Maladies infectieuses pediatriques a prevention vaccinale. Tendances et developpements en Belgique et dans les Communautes. Institut Scientifique de Sante Publique. 2012 (https://epidemio.wiv-isp.be/ID/diseases/Documents/VPD_Rapport_2012_FR.pdf).

[42] Gabutti G, The seroepidemiology of varicella in Italy. Epidemiology & Infection 2001; 126(3): 433–440. 11467800 10.1017/s0950268801005398 PMC2869711

[43] Baldo V, Varicella: epidemiological aspects and vaccination coverage in the Veneto Region. BMC Infectious Diseases 2009; 9: 150. 19737419 10.1186/1471-2334-9-150 PMC2751773

[44] Perez-Farinos N, Varicella and herpes zoster in Madrid, based on the Sentinel General Practitioner Network: 1997–2004. BMC Infectious Diseases 2007; 7: 59. 17570859 10.1186/1471-2334-7-59 PMC1913920

[45] Bramley JC, Jones IG. Epidemiology of chickenpox in Scotland: 1981 to 1998. Communicable Disease and Public Health 2000; 3(4): 282–287. 11280260

[46] Lipke M, Paradowska-Stankiewicz I. Chickenpox in Poland in 2011. Przeglad Epidemiologiczny 2013; 67(2): 195–197, 317–198. 24040715

[47] Rogalska J, Paradowska-Stankiewicz I. Chickenpox in Poland in 2012. Przeglad Epidemiologiczny 2014; 68(2): 201–204, 323–204. 25135499

[48] Anon. Réseau Sentinelles. Bilan annuel 2008–2014 (https://websenti.u707.jussieu.fr/sentiweb/?rub=39).

[49] de Melker H, The epidemiology of varicella and herpes zoster in the Netherlands: implications for varicella zoster virus vaccination. Vaccine 2006; 24(18): 3946–3952. 16564115 10.1016/j.vaccine.2006.02.017

[50] Pierik JG, Epidemiological characteristics and societal burden of varicella zoster virus in the Netherlands. BMC Infectious Diseases 2012; 12: 110. 22574722 10.1186/1471-2334-12-110 PMC3464966

