# Supplementary material for: A systematic review of varicella seroprevalence in European countries before universal childhood immunization: deriving incidence from seroprevalence data
Source: Epidemiol Infect. 2017 Aug 22;145(13):2666–77. doi: 10.1017/S0950268817001546 (PMC5647669; doi:10.1017/S0950268817001546)
Supplement: Supplementary file 1 [file S0950268817001546sup.zip › S0950268817001546sup001.docx]

**Table S.1.** Characteristics of the studies (n=43) on varicella epidemiology in European countries before universal childhood immunization that were included in the analyses.

| **First Author, year of publication** | **Country** | **Years of data collection** | **Population and method of sera collection** | **Age range/mean age*** | **Sample size** | **Laboratory method** | **Diagnostic test** | **Equivocal results** | **Ref** |
| --- | --- | --- | --- | --- | --- | --- | --- | --- | --- |
| Leuridan, 2011 | Belgium | 2006 - 2008 | Pregnant women, population-based | 18 - 40 y | 213 | ELISA | Enzygnost | NP | [1] |
| Thiry, 2002 | Belgium | 1999 - 2000 | General, residual sera | 1 - 44 y | 1673 | ELISA | Enzygnost | Reported | [2] |
| Vandersmissen, 2000 | Belgium | 1996 - 1997 | Health care staff, population-based | 37.2 y* | 4923 | ELISA | Enzygnost | Re-tested | [3] |
| Alanen, 2005 | Finland | 2000 | Pregnant women, population-based | 16 - 45 y | 558 | ELISA | In - house | NP | [4] |
| Koskiniemi, 2007 | Finland | 1995 - 1996 | Hospitalized patients with suspected viral infection, population-based | 0 - 80 y | 2842 | ELISA | In - house | NP | [5] |
| Nardone, 2007 | Finland | 1997 - 1998 | General, residual sera | 1 -29 y | 2123 | ELISA | Enzygnost | Considered positives after validation by FAMA | [6] |
| Khoshnood 2006 | France | 2003 - 2004 | General, residual sera | 1 - 30 y | 1257 | ELISA | Trinity Biotech | Re-tested and reported | [7] |
| Pinquier, 2009 | France | 2005 - 2007 | Children with a scheduled blood sample, population based | 0 - 15 m | 345 | TRFIA | In - house | NP | [8] |
| Saadatian-Elahi, 2007 | France | 2005 | Pregnant women, population-based | 19 - 43 y | 486 | ELISA | Enzygnost | NP | [9] |
| Sauerbrei, 2004 | Germany | 1995 - 1996 | Pregnant women, population-based | 19 - 37 y | 215 | IFAT | In - house | NP | [10] |
| Wutzler, 2001 | Germany | 1995 - 1999 | General, residual sera | 0 - 70+ y | 4602 | ELISA | Enzygnost | Re-tested | [11] |
| Kavaliotis, 2003 | Greece | 1999 - 2001 | Children hospitalized, population-based | 1 - 13 y | 632 | ELISA | Genzyme Virotech | NP | [12] |
| Thorarinsdottir, 2009 | Iceland | 2001 - 2002 | General, residual sera | 0 - 18 y | 280 | ELISA | NP | Re-tested and excluded | [13] |
| Knowles, 2004 | Ireland | 2002 | Pregnant women, population-based | 20 – 24 y | 4794 | ELISA | Diamedix | Reported | [14] |
| Nardone, 2007 | Ireland | 2003 | General, residual sera | 1 -29 y | 1575 | ELISA | Diamedix | Considered positives after validation by FAMA | [6] |
| Alfonsi, 2007 | Italy | 2001 - 2002 | Women reproductive age, residual se | 17 - 42 y | 728 | ELISA | Vircell | Re-tested and reported | [15] |
| Fedeli, 2002 | Italy | 1998 - 2001 | Health care staff, population-based | 23 - 60 y | 333 | ELISA | Enzygnost | Reported | [16] |
| Gabutti, 2001 | Italy | 1996 - 1997 | General, residual sera | 0 - 60+ y | 3179 | ELISA | Enzygnost | NP | [17] |
| Gabutti, 2008 | Italy | 2003 - 2004 | General, residual sera | 0 - 50 y | 3094 | ELISA | Enzygnost | Re-tested and reported | [18] |
| Guido, 2012 | Italy | 2008 - 2009 | General, population-based | 15 - 49 y | 539 | ELISA | Enzygnost | Reported | [19] |
| Trevisan, 2006 | Italy | 2004 | Health care students, population-based | 23.4 y* | 1024 | ELISA | Enzygnost | Considered negative | [20] |
| Trevisan, 2007 | Italy | 2003 - 2005 | Health care students, population-based | 22.9 y* | 616 | ELISA | Enzygnost | Considered negative | [21] |
| Mossong, 2004 | Luxembourg | 2000 - 2002 | General, population-based | 4 y - 60+y | 2679 | ELISA | Enzygnost | Fixed cut off pos/neg used | [22] |
| de Melker, 2006 | Netherlands | 1995 - 1996 | General, population-based | 3m - 79 y | 2038 | ELISA | Human | NP | [23] |
| van Lier, 2013 | Netherlands | 2006 - 2007 | General, population-based | 0 - 79 y | 6383 | RT-PCR | Luminex | Fixed cut off pos/neg used | [24] |
| van Rijckevorsel, 2012 | Netherlands | 2004 | General, population-based | 17 - 90 y | 1341 | ELISA | Euroimmun | Considered negative | [25] |
| Siennicka, 2009 | Poland | 1995 - 2004 | General, population-based | 1 - 19 y | 1268 | ELISA | Enzygnost | Re-tested and excluded | [26] |
| Nardone, 2007 | Slovakia | 2002 | General, population-based | 1 -29 y | 2511 | ELISA | Euroimmun | Considered positives after validation by FAMA | [6] |
| Socan, 2010 | Slovenia | 2006 | General, residual sera | 6 m - 60+ y | 3689 | ELISA | Enzygnost | Re-tested and considered negative | [27] |
| de Juanes, 2005 | Spain | 2003 | Health care staff and students, population-based | 18 - 40 y | 269 | ELISA | Enzygnost | NP | [28] |
| Diez Domingo, 2005 | Spain | 2003 | General, population-based | 6 - 15 y | 184 | ELISA | Enzygnost | NP | [29] |
| Gil, 1998 | Spain | 1993 | Students, population-based | 14 - 17 y | 1191 | ELISA | Menarini | NP | [30] |
| ISCIII - CNE, 2000 | Spain | 1996 | General (attending health centres), population-based | 2 - 39 y | 3687 | ELISA | Enzygnost | Re-tested and considered positive | [31] |
| Perez-Farinos, 2008 | Spain | 1999 - 2000 | General (attending blood extraction centres), population-based | 2 - 40 y | 1829 | ELISA | Enzygnost | NP | [32] |
| Plans, 2007 | Spain | 2003 | Pregnant women, population-based | 15 - 49 y | 1522 | ELISA | Vircell | NP | [33] |
| Salleras, 2000 | Spain | 1996 | General, population-based | 6 - 65+ y | 2136 | ELISA | Enzygnost | NP | [34] |
| Salleras, 2008 | Spain | 2002 | General, population-based | 5 - 65+ y | 2619 | ELISA | Enzygnost | NP | [35] |
| Aebi, 2001 | Switzerland | 1997 - 1998 | General (hospital inpatients and outpatients), population-based | 0 - 16 y | 927 | IIF | In - house | NP | [36] |
| Baer, 2005 | Switzerland | 1999 - 2003 | Health care students, population-based | 22 - 48 y | 149 | ELISA | Enzygnost | Re-tested and considered negative | [37] |
| Heininger, 2001 | Switzerland | 1992 - 93 1995 - 96 | General, population-based | 6 - 15 y | 1709 | ELISA | Enzygnost | Reported | [38] |
| Heininger, 2005 | Switzerland | 1999 - 2000 | General (hospital inpatients), population-based | 1 - 18 y | 449 | ELISA | Enzygnost | Re-tested and reported | [39] |
| Heininger, 2006 | Switzerland | 1994 - 1999 | General (hospital inpatients), population-based | 0 - 16 m | 253 | ELISA | Enzygnost | Re-tested and considered negative | [40] |
| Kudesia,2002 | UK | 1992 | General, residual sera | 1 - 39 y | 262 | ELISA | Bio-Stat | NP | [41] |
| Nardone, 2007 | UK | 1996 | General, residual | 0 - 29 y | 2091 | ELISA | Diamedix | Considered positives after validation by FAMA | [6] |
| Pembrey, 2013 | UK | 2008 | Pregnant women, population based | 20 – 34 y | 949 | ELISA | Diasorin | Reported | [42] |
| Talukder, 2007 | UK | 2001 - 2002 | Pregnant women (born in the UK), population-based | 28 y* | 266 | ELISA | Enzygnost/Diamedix | NP | [43] |

1. Leuridan E, et al. Kinetics of maternal antibodies against rubella and varicella in infants. Vaccine. 2011;29(11):2222-6. Epub 2010/06/19. doi: 10.1016/j.vaccine.2010.06.004. PubMed PMID: 20558248.

2. Thiry N, et al. The seroepidemiology of primary varicella-zoster virus infection in Flanders (Belgium). European journal of pediatrics. 2002;161(11):588-93. Epub 2002/11/09. doi: 10.1007/s00431-002-1053-2. PubMed PMID: 12424583.

3. Vandersmissen G, et al. Occupational risk of infection by varicella zoster virus in Belgian healthcare workers: a seroprevalence study. Occupational and environmental medicine. 2000;57(9):621-6. Epub 2000/08/10. PubMed PMID: 10935943; PubMed Central PMCID: PMCPmc1740019.

4. Alanen A, et al. Seroprevalence, incidence of prenatal infections and reliability of maternal history of varicella zoster virus, cytomegalovirus, herpes simplex virus and parvovirus B19 infection in South-Western Finland. BJOG : an international journal of obstetrics and gynaecology. 2005;112(1):50-6. Epub 2005/01/25. doi: 10.1111/j.1471-0528.2004.00320.x. PubMed PMID: 15663397.

5. Koskiniemi M, et al. Genotypic analysis of varicella-zoster virus and its seroprevalence in Finland. Clinical and vaccine immunology : CVI. 2007;14(9):1057-61. Epub 2007/07/13. doi: 10.1128/cvi.00348-06. PubMed PMID: 17626161; PubMed Central PMCID: PMCPmc2043318.

6. Nardone A, et al. The comparative sero-epidemiology of varicella zoster virus in 11 countries in the European region. Vaccine. 2007;25(45):7866-72. doi: 10.1016/j.vaccine.2007.07.036. PubMed PMID: 17919788.

7. Khoshnood B, et al. Seroprevalence of varicella in the French population. The Pediatric infectious disease journal. 2006;25(1):41-4. Epub 2006/01/06. PubMed PMID: 16395101.

8. Pinquier D, et al. Prevalence of anti-varicella-zoster virus antibodies in French infants under 15 months of age. Clinical and vaccine immunology : CVI. 2009;16(4):484-7. Epub 2009/01/30. doi: 10.1128/cvi.00397-08. PubMed PMID: 19176690; PubMed Central PMCID: PMCPmc2668269.

9. Saadatian-Elahi M, et al. Seroprevalence of varicella antibodies among pregnant women in Lyon-France. European journal of epidemiology. 2007;22(6):405-9. Epub 2007/05/31. doi: 10.1007/s10654-007-9136-z. PubMed PMID: 17534728.

10. Sauerbrei A, et al. [Antibodies against vaccine-preventable diseases in pregnant women and their offspring. Measles, mumps, rubella, poliomyelitis, and varicella]. Bundesgesundheitsblatt, Gesundheitsforschung, Gesundheitsschutz. 2004;47(1):10-5. Epub 2004/06/19. doi: 10.1007/s00103-003-0689-z. PubMed PMID: 15205818.

11. Wutzler P, et al. Seroprevalence of varicella-zoster virus in the German population. Vaccine. 2001;20(1-2):121-4. Epub 2001/09/25. PubMed PMID: 11567755.

12. Kavaliotis J, et al. How reliable is the history of chickenpox? Varicella serology among children up to 14 years of age. Int J Infect Dis. 2003;7(4):274-7. Epub 2003/12/06. PubMed PMID: 14656418.

13. Thorarinsdottir H, et al. [Varicella in Icelandic children--epidemiology and complications]. Laeknabladid. 2009;95(2):113-8. Epub 2009/02/07. PubMed PMID: 19197109.

14. Knowles SJ, et al. Susceptibility to infectious rash illness in pregnant women from diverse geographical regions. Communicable disease and public health / PHLS. 2004;7(4):344-8. Epub 2005/03/23. PubMed PMID: 15779804.

15. Alfonsi V, et al. Susceptibility to varicella in childbearing age women, Central Italy: is there a need for vaccinating this population group? Vaccine. 2007;25(32):6086-8. Epub 2007/07/17. doi: 10.1016/j.vaccine.2007.05.019. PubMed PMID: 17629374.

16. Fedeli U, et al. Susceptibility of healthcare workers to measles, mumps rubella and varicella. The Journal of hospital infection. 2002;51(2):133-5. Epub 2002/07/02. PubMed PMID: 12090801.

17. Gabutti G, et al. The seroepidemiology of varicella in Italy. Epidemiol Infect. 2001;126(3):433-40. Epub 2001/07/27. PubMed PMID: 11467800; PubMed Central PMCID: PMCPmc2869711.

18. Gabutti G, et al. The epidemiology of Varicella Zoster Virus infection in Italy. BMC Public Health. 2008;8:372. doi: 10.1186/1471-2458-8-372. PubMed PMID: 18954432; PubMed Central PMCID: PMCPMC2601043.

19. Guido M, et al. Susceptibility to varicella-zoster among pregnant women in the province of Lecce, Italy. Journal of clinical virology : the official publication of the Pan American Society for Clinical Virology. 2012;53(1):72-6. Epub 2011/11/15. doi: 10.1016/j.jcv.2011.10.007. PubMed PMID: 22074933.

20. Trevisan A, et al. Prevalence of childhood exanthematic disease antibodies in paramedical students: need of vaccination. Vaccine. 2006;24(2):171-6. Epub 2005/09/13. doi: 10.1016/j.vaccine.2005.07.062. PubMed PMID: 16154242.

21. Trevisan A, et al. Immunity against infectious diseases: predictive value of self-reported history of vaccination and disease. Infection control and hospital epidemiology. 2007;28(5):564-9. Epub 2007/04/28. doi: 10.1086/516657. PubMed PMID: 17464916.

22. Mossong J, et al. Seroprevalence and force of infection of varicella-zoster virus in Luxembourg. Epidemiol Infect. 2004;132(6):1121-7. Epub 2005/01/08. PubMed PMID: 15635970; PubMed Central PMCID: PMCPmc2870204.

23. de Melker H, et al. The epidemiology of varicella and herpes zoster in The Netherlands: implications for varicella zoster virus vaccination. Vaccine. 2006;24(18):3946-52. Epub 2006/03/28. doi: 10.1016/j.vaccine.2006.02.017. PubMed PMID: 16564115.

24. van Lier A, et al. Varicella zoster virus infection occurs at a relatively young age in The Netherlands. Vaccine. 2013;31(44):5127-33. Epub 2013/08/27. doi: 10.1016/j.vaccine.2013.08.029. PubMed PMID: 23973248.

25. van Rijckevorsel GG, et al. Seroprevalence of varicella-zoster virus and predictors for seronegativity in the Amsterdam adult population. BMC infectious diseases. 2012;12:140. Epub 2012/06/23. doi: 10.1186/1471-2334-12-140. PubMed PMID: 22721551; PubMed Central PMCID: PMCPmc3434062.

26. Siennicka J, et al. Seroprevalence of varicella-zoster virus in Polish population. Przeglad epidemiologiczny. 2009;63(4):495-9. Epub 2010/02/04. PubMed PMID: 20120946.

27. Socan M, et al. Varicella susceptibility and transmission dynamics in Slovenia. BMC Public Health. 2010;10:360. Epub 2010/06/25. doi: 10.1186/1471-2458-10-360. PubMed PMID: 20573202; PubMed Central PMCID: PMCPmc2901375.

28. De Juanes JR, et al. Seroprevalence of varicella antibodies in healthcare workers and health sciences students. Reliability of self-reported history of varicella. Vaccine. 2005;23(12):1434-6. Epub 2005/01/27. doi: 10.1016/j.vaccine.2004.10.003. PubMed PMID: 15670877.

29. Diez-Domingo J, et al. Seroprevalence of varicella among children and adolescents in Valencia, Spain. Reliability of the parent's reported history and the medical file for identification of potential candidates for vaccination. Human vaccines. 2005;1(5):204-6. Epub 2006/10/03. PubMed PMID: 17012857.

30. Gil A, et al. Prevalence of antibodies against varicella zoster, herpes simplex (types 1 and 2), hepatitis B and hepatitis A viruses among Spanish adolescents. The Journal of infection. 1998;36(1):53-6. Epub 1998/11/20. PubMed PMID: 9515669.

31. Instituto de Salud Carlos III. Centro Nacional de Epidemiología. Estudio seroepidemiológico: situación de las enfermedades vacunables en España. Available from: <http://www.isciii.es/ISCIII/es/contenidos/fd-servicios-cientifico-tecnicos/fd-vigilancias-alertas/fd-enfermedades/SEROEPIDEMIOLOGICO.pdf>.

32. Perez-Farinos N, et al. Seroprevalence of antibodies to varicella-zoster virus in Madrid (Spain) in the absence of vaccination. Central European journal of public health. 2008;16(1):41-4. Epub 2008/05/08. PubMed PMID: 18459480.

33. Plans P, et al. Prevalence of varicella-zoster antibodies in pregnant women in Catalonia (Spain). Rationale for varicella vaccination of women of childbearing age. BJOG : an international journal of obstetrics and gynaecology. 2007;114(9):1122-7. Epub 2007/08/02. doi: 10.1111/j.1471-0528.2007.01454.x. PubMed PMID: 17666097.

34. Salleras L, et al. Seroepidemiology of varicella-zoster virus infection in Catalonia (Spain). Rationale for universal vaccination programmes. Vaccine. 2000;19(2-3):183-8. Epub 2000/08/10. PubMed PMID: 10930671.

35. Salleras L, et al. Seroprevalence of varicella zoster virus infection in child and adult population of Catalonia (Spain). Medical microbiology and immunology. 2008;197(3):329-33. Epub 2007/11/16. doi: 10.1007/s00430-007-0064-z. PubMed PMID: 18004592.

36. Aebi C, et al. Age-specific seroprevalence to varicella-zoster virus: study in Swiss children and analysis of European data. Vaccine. 2001;19(23-24):3097-103. Epub 2001/04/20. PubMed PMID: 11312004.

37. Baer G, et al. Seroprevalence and immunization history of selected vaccine preventable diseases in medical students. Vaccine. 2005;23(16):2016-20. Epub 2005/03/01. doi: 10.1016/j.vaccine.2004.03.073. PubMed PMID: 15734076.

38. Heininger U, et al. Seroprevalence of varicella-zoster virus immunoglobulin G antibodies in Swiss adolescents and risk factor analysis for seronegativity. The Pediatric infectious disease journal. 2001;20(8):775-8. Epub 2001/12/06. PubMed PMID: 11734740.

39. Heininger U, et al. Reliability of varicella history in children and adolescents. Swiss medical weekly. 2005;135(17-18):252-5. Epub 2005/06/21. doi: 2005/17/smw-11007. PubMed PMID: 15965827.

40. Heininger U, et al. Seroprevalence of Varicella-Zoster virus IgG antibodies in Swiss children during the first 16 months of age. Vaccine. 2006;24(16):3258-60. Epub 2006/02/07. doi: 10.1016/j.vaccine.2006.01.026. PubMed PMID: 16459000.

41. Kudesia G, et al. Changes in age related seroprevalence of antibody to varicella zoster virus: impact on vaccine strategy. J Clin Pathol. 2002;55(2):154-5. PubMed PMID: 11865016; PubMed Central PMCID: PMCPMC1769589.

42. Pembrey L, et al. Seroprevalence of cytomegalovirus, Epstein Barr virus and varicella zoster virus among pregnant women in Bradford: a cohort study. PloS one. 2013;8(11):e81881. Epub 2013/12/07. doi: 10.1371/journal.pone.0081881. PubMed PMID: 24312372; PubMed Central PMCID: PMCPmc3842274.

43. Talukder YS, et al. The seroepidemiology of varicella zoster virus among pregnant Bangladeshi and white British women in the London Borough of Tower Hamlets, UK. Epidemiol Infect. 2007;135(8):1344-53. Epub 2007/04/21. doi: 10.1017/s0950268807008497. PubMed PMID: 17445317; PubMed Central PMCID: PMCPmc2870708.
