# Supplementary material for: A systematic review of varicella seroprevalence in European countries before universal childhood immunization: deriving incidence from seroprevalence data
Source: Epidemiol Infect. 2017 Aug 22;145(13):2666–77. doi: 10.1017/S0950268817001546 (PMC5647669; doi:10.1017/S0950268817001546)
Supplement: Supplementary file 1 [file S0950268817001546sup.zip › S0950268817001546sup002.docx]

**Table S.2:** Age-specific forces of infection based on the piecewise constant catalytic model.

|  | Age-specific forces of infection (95% CI) | | |
| --- | --- | --- | --- |
|  | <5 yrs | 5-9 yrs | >=10 yrs |
| Belgium | 0.26 [0.24;0.29] | 0.3 [0.2;0.42] | 0.05 [0.04;0.07] |
| Finland | 0.14 [0.13;0.16] | 0.49 [0.44;0.55] | 0.01 [0;0.02] |
| France | 0.22 [0.2;0.25] | 0.26 [0.17;0.38] | 0.07 [0.04;0.11] |
| Germany | 0.18 [0.16;0.2] | 0.46 [0.39;0.54] | 0.05 [0.04;0.07] |
| Greece | 0.09 [0.07;0.1] | 0.19 [0.1;0.29] | 0.14 [0;0.5] |
| Iceland | 0.017 [0.11;0.2] | 0.62 [0.5;1.12] | 0.0 [0.0;0.0] |
| Ireland | 0.18 [0.15;0.22] | 0.36 [0.25;0.43] | 0.01 [0;0.04] |
| Italy | 0.1 [0.09;0.12] | 0.26 [0.21;0.3] | 0.05 [0.04;0.07] |
| Luxembourg | 0.31 [0.26;0.37] | 0.29 [0.17;0.41] | 0.03 [0.02;0.06] |
| Netherlands | 0.33 [0.3;0.36] | 0.42 [0.34;0.49] | 0.01 [0;0.02] |
| Poland | 0.12 [0.1;0.14] | 0.27 [0.2;0.35] | 0.14 [0.06;0.23] |
| Slovakia | 0.13 [0.11;0.14] | 0.34 [0.27;0.43] | 0.09 [0.06;0.14] |
| Slovenia | 0.17 [0.15;0.19] | 0.4 [0.33;0.48] | 0.04 [0.03;0.06] |
| Spain | 0.16 [0.14;0.17] | 0.36 [0.32;0.4] | 0.04 [0.03;0.05] |
| Switzerland | 0.09 [0.08;0.11] | 0.66 [0.6;0.72] | 0.02 [0;0.09] |
| UK | 0.21 [0.19;0.23] | 0.22 [0.15;0.29] | 0.04 [0.02;0.06] |
